# Linking Hypothesis and Number of Response Options Modulate Inferred Scalar Implicature Rate

**DOI:** 10.3389/fpsyg.2019.00189

**Published:** 2019-02-12

**Authors:** Masoud Jasbi, Brandon Waldon, Judith Degen

**Affiliations:** ^1^Department of Linguistics, Harvard University, Cambridge, MA, United States; ^2^Department of Linguistics, Stanford University, Stanford, CA, United States

**Keywords:** scalar implicature, methodology, linking hypothesis, experimental pragmatics, truth value judgment task

## Abstract

The past 15 years have seen increasing experimental investigations of core pragmatic questions in the ever more active and lively field of experimental pragmatics. Within experimental pragmatics, many of the core questions have relied on the operationalization of the theoretical notion of “implicature rate.” Implicature rate based results have informed the work on acquisition, online processing, and scalar diversity, inter alia. Implicature rate has typically been quantified as the proportion of “pragmatic” judgments in two-alternative forced choice truth value judgment tasks. Despite its theoretical importance, this linking hypothesis from implicature rate to behavioral responses has never been extensively tested. Here we show that two factors dramatically affect the “implicature rate” inferred from truth value judgment tasks: (a) the number of responses provided to participants; and (b) the linking hypothesis about what constitutes a “pragmatic” judgment. We argue that it is time for the field of experimental pragmatics to engage more seriously with its foundational assumptions about how theoretical notions map onto behaviorally measurable quantities, and present a sketch of an alternative linking hypothesis that derives behavior in truth value judgment tasks from probabilistic utterance expectations.

## 1. Introduction

The past 15 years have seen the rise and development of a bustling and exciting new field at the intersection of linguistics, psychology, and philosophy: *experimental pragmatics* (Chierchia et al., 2001; Noveck and Posada, 2003; Bott and Noveck, 2004; Papafragou and Tantalou, 2004; Breheny et al., 2006, 2013; De Neys and Schaeken, 2007; Noveck and Reboul, 2008; Bonnefon et al., 2009; Geurts and Pouscoulous, 2009; Huang and Snedeker, 2009; Grodner et al., 2010; Barner et al., 2011; Katsos and Bishop, 2011; Tomlinson et al., 2013; Degen and Tanenhaus, 2015, 2016; Bott and Chemla, 2016; van Tiel et al., 2016). Experimental pragmatics is devoted to experimentally testing theories of how language is used in context. How do listeners draw inferences about the – often underspecified – linguistic signal they receive from speakers? How do speakers choose between the many utterance alternatives they have at their disposal?

The most prominently studied phenomenon in experimental pragmatics is undoubtedly *scalar implicature*. Scalar implicatures arise as a result of a speaker producing the weaker of two ordered scalemates (Horn, 1972; Grice, 1975; Hirschberg, 1985; Geurts, 2010). Examples are provided in (1-2).

(1) Some of her pets are cats.
     *Implicature:* Some, but not all, of her pets are cats.
     *Scale:* 〈all, some〉
(2) She owns a cat or a dog.
     *Implicature:* She owns a cat or a dog, but not both.
     *Scale:* 〈and, or〉

A listener, upon observing the utterances in (1-2) typically infers that the speaker intended to convey the meanings listed as *Implicature*s, respectively. Since Grice (1975), the agreed-upon abstract rationalization the listener could give for their inference goes something like this: the speaker could have made a more informative statement by producing the stronger alternative (e.g., *All of her pets are cats* in (1)). If the stronger alternative is true, they should have produced it to comply with the Cooperative Principle. They chose not to. Assuming the speaker knows whether the stronger alternative is true, it must not be true. The derivation procedure for *ad hoc* exhaustivity inferences such as in (3) is assumed to be calculable in the same way as for scalar implicatures, though the scale is assumed to be contextually driven.

(3) She owns a cat.
     *Implicature:* She owns only a cat.
     *Scale:* 〈cat and dog, cat〉

Because the basic reconstruction of the inference is much more easily characterized for scalar implicatures than for other implicatures, scalar implicatures have served as a test bed for many questions in experimental pragmatics, including, but not limited to:

1. Are scalar inferences default inferences, in the sense that they arise unless blocked by (marked) contexts (Horn, 1984; Levinson, 2000; Degen, 2015)?
2. Are scalar inferences default inferences, in the sense that they are computed automatically in online processing and only canceled in a second effortful step if required by context (Bott and Noveck, 2004; Breheny et al., 2006; Huang and Snedeker, 2009; Grodner et al., 2010; Politzer-Ahles and Fiorentino, 2013; Tomlinson et al., 2013; Degen and Tanenhaus, 2016)?
3. What are the (linguistic and extra-linguistic) factors that affect whether a scalar implicature is derived (Breheny et al., 2006, 2013; De Neys and Schaeken, 2007; Bonnefon et al., 2009; Zondervan, 2010; Chemla and Spector, 2011; Bergen and Grodner, 2012; Degen and Goodman, 2014; Degen, 2015; Degen and Tanenhaus, 2015, 2016; Potts et al., 2015; de Marneffe and Tonhauser, in press)?
4. How much diversity is there across implicature types, and within scalar implicatures across scale types, in whether or not an implicature is computed (Doran et al., 2012; van Tiel et al., 2016)?
5. At what age do children acquire the ability to compute implicatures (Noveck, 2001; Musolino, 2004; Papafragou and Tantalou, 2004; Barner et al., 2011; Katsos and Bishop, 2011; Stiller et al., 2015; Horowitz et al., 2017)?

In addressing all of these questions, it has been important to obtain estimates of *implicature rates*. For 1., implicature rates from experimental tasks can be taken to inform whether scalar implicatures should be considered default inferences. For 2., processing measures on responses that indicate implicatures can be compared to processing measures on responses that indicate literal interpretations. For 3., contextual effects can be examined by comparing implicature rates across contexts. For 4., implicature rates can be compared across scales (or across implicature types). For 5., implicature rates can be compared across age groups.

A standard measure that has stood as a proxy for implicature rate across many studies is the proportion of “pragmatic” judgments in truth value judgment paradigms (Noveck, 2001; Noveck and Posada, 2003; Bott and Noveck, 2004; De Neys and Schaeken, 2007; Geurts and Pouscoulous, 2009; Chemla and Spector, 2011; Degen and Goodman, 2014; Degen and Tanenhaus, 2015). In these kinds of tasks, participants are provided a set of facts, either presented visually or via their own knowledge of the world. They are then asked to judge whether a sentence intended to describe those facts is true or false (or alternatively, whether it is right or wrong, or they are asked whether they agree or disagree with the sentence). The crucial condition for assessing implicature rates in these kinds of studies typically consists of a case where the facts are such that the stronger alternative is true and the target utterance is thus also true but underinformative. For instance, Bott and Noveck (2004) asked participants to judge sentences like “Some elephants are mammals”, when world knowledge dictates that all elephants are mammals. Similarly, Degen and Tanenhaus (2015) asked participants to judge sentences like “You got some of the gumballs” in situations where the visual evidence indicated that the participant received all the gumballs from a gumball machine. In these kinds of scenarios, the story goes, if a participant responds “FALSE”, that indicates that they computed a scalar implicature, e.g., to the effect of “Not all elephants are mammals” or “You didn't get all of the gumballs”, which is (globally or contextually) false. If instead a participant responds “TRUE”, that is taken to indicate that they interpreted the utterance literally as “Some, and possibly all, elephants are mammals” or “You got some, and possibly all, of the gumballs”.

Using the proportion of “FALSE” responses on true but underinformative trials as a proxy for implicature rate is common in experimental pragmatics. For example, in one of the first studies to investigate scalar implicatures experimentally, Noveck (2001) tested adults' and children's interpretations of the scalar items *might* and *some*. The dependent measure in Noveck (2001) was the rate of “logically correct responses,” i.e., responding “yes” to statements such as *Some giraffes have long necks* or *There might be a parrot [in the box]* when there had to be a parrot in the box. He found that children responded “yes” more frequently than adults, and concluded that children interpret scalar items *some* and *might* more logically (i.e., literally). Similarly in another landmark study, Papafragou and Musolino (2003) tested children and adults interpretation of the following set of scalar items: <*two, three*>, <*some, all*>, and <*finish, start*>. The dependent measure in this study was the proportion of “No” responses to a puppet's underinformative statement. The study concluded that “while adults overwhelmingly rejected infelicitous descriptions, children almost never did so.” Furthermore, the study compared implicature rates across scales and concluded that “children also differed from from adults in that their rejection rate on the numerical scale was reliably higher than on the two other scales.” In their final experiment, Papafragou and Musolino (2003) modified their task to invite scalar inferences more easily. They reported that this manipulation resulted in a significantly higher rejection rates. Based on these results, they concluded that children's ability to compute implicatures is affected by the type of scalar item as well as children's awareness of the task's goals. Since these early pioneering studies, the rate of “FALSE” (or “No,” “Wrong,” “Disagree”) responses on underinformative trials in truth-value judgment tasks has become a commonplace dependent measure (Geurts and Pouscoulous, 2009; Doran et al., 2012; Potts et al., 2015, inter alia.)

Given the centrality of the theoretical notion of “implicature rate” to much of experimental pragmatics, there is to date a surprising lack of discussion of the basic assumption that it is adequately captured by the proportion of “FALSE” responses in truth value judgment tasks [but see Geurts and Pouscoulous, 2009; Katsos and Bishop, 2011; Benz and Gotzner, 2014; Degen and Goodman, 2014; Sikos et al., 2019]. Indeed, the scalar implicature acquisition literature was shaken up when Katsos and Bishop (2011) showed that simply by introducing an additional response option, children started looking much more pragmatic than had been previously observed in a binary judgment paradigm. Katsos and Bishop (2011) allowed children to distribute a small, a big, or a huge strawberry to a puppet depending on “how good the puppet said it.” The result was that children gave on average smaller strawberries to the puppet when he produced underinformative utterances compared to when he produced literally true and pragmatically felicitous utterances, suggesting that children do, in fact, display pragmatic ability even at ages when they had previously appeared not to.

But this raises an important question: in truth value judgment tasks, how does the researcher know whether an interpretation is literal or the result of an implicature computation? The binary choice task typically used is appealing in part because it allows for a direct mapping from response options—“TRUE” and “FALSE'—to interpretations—literal and pragmatic. That the seeming simplicity of this mapping is illusory becomes apparent once a third response option is introduced, as in the Katsos and Bishop (2011) case. How is the researcher to interpret the intermediate option? Katsos and Bishop (2011) grouped the intermediate option with the negative endpoint of the scale for the purpose of categorizing judgments as literal vs. pragmatic, i.e., they interpreted the intermediate option as pragmatic. But it seems just as plausible that they could have grouped it with the positive endpoint of the scale and taken the hard line that only truly “FALSE” responses constitute evidence of a full-fledged implicature. The point here is that there has been remarkably little consideration of *linking hypotheses* between behavioral measures and theoretical constructs in experimental pragmatics, a problem in many subfields of psycholinguistics (Tanenhaus, 2004). We argue that it is time to engage more seriously with these issues.

We begin by reporting an experiment that addresses the following question: do the number of response options provided in a truth value judgment task and the way that responses are grouped into pragmatic (“SI”) and literal (“no SI”) change inferences about scalar implicature rates? Note that this way of asking the question assumes two things: first, that whatever participants are doing in a truth value judgment task, the behavioral measure can be interpreted as providing a measure of interpretation; and second, that listeners either do or do not compute an implicature on any given occasion. In the General Discussion we will discuss both of these issues. Following Degen and Goodman (2014), we will offer some remarks on why truth value judgment tasks are better thought of as measuring participants' estimates of speakers' *production* probabilities. This will suggest a completely different class of linking hypotheses. We then discuss an alternative conception of scalar implicature as a probabilistic phenomeonen, a view that has recently rose to prominence in the subfield of probabilistic pragmatics (Franke and Jäger, 2016; Goodman and Frank, 2016). This alternative conception of scalar implicature, we argue, affords developing and testing quantitative linking hypotheses in a rigorous and motivated way.

**Figure 1:**
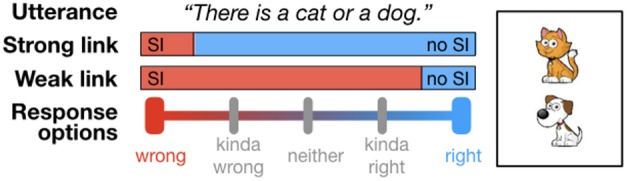
Strong and Weak link from response options to researcher inference about scalar implicature rate, exemplified for the disjunctive utterance when the conjunction is true.

Consider a setup in which a listener is presented a card with a depiction of either one or two animals (see Figure 1 for an example). As in a standard truth value judgment task, the listener then observes an underinformative utterance about this card (e.g., “There is a cat or a dog on the card”) and is asked to provide a judgment on a scale with 2, 3, 4, or 5 response options, with endpoints “wrong” and “right.”^1^ In the binary case, this reproduces the standard truth value judgment task. Figure 1 exemplifies (some of) the researcher's options for grouping responses. Under what we will call the “Strong link” assumption, only the negative endpoint of the scale is interpreted as evidence for a scalar implicature having been computed. Under the “Weak link” assumption, in contrast, any response that does not correspond to the positive endpoint of the scale is interpreted as evidence for a scalar implicature having been computed. Intermediate grouping schemes are also possible, but these are the ones we will consider here. Note that for the binary case, the Weak and Strong link return the same categorization scheme, but for any number of response options greater than 2, the Weak and Strong link can in principle lead to differences in inferences about implicature rate.

Let's examine an example. Assume three response options (wrong, neither, right). Assume further that each of the three responses was selected by a third of participants, i.e., the distributions of responses is 1/3, 1/3, and 1/3. Under the Strong link, we infer that this task yielded an implicature rate of 2/3. Under the Weak link, we infer that this task yielded an implicature rate of 1/3. This is quite a drastic difference if we are, for instance, interested in whether scalar implicatures are inference defaults and we would like to interpret an implicature rate of above an arbitrary threshold (e.g., 50%) as evidence for such a claim. Under the Strong link, we would conclude that scalar implicatures are not defaults. Under the Weak link, we would conclude that they are. In the experiment reported in the following section, we presented participants with exactly this setup. We manipulated the number of response options between participants and analyzed the results under different linking hypothesis^2^.

## 2. Experiment

Participants played an online card game in which they were asked to judge descriptions of the contents of cards. Different groups of participants were presented with different numbers of response options. On critical trials, participants were presented with descriptions for the cards that typically result in exhaustivity implicatures (“There is a cat on the card” when there was a cat and a dog) or scalar implicatures (“There is a cat or a dog on the card” when there was a cat and a dog). We categorized their responses on such trials according to the Weak and the Strong link introduced above, and tested whether the number of response options and the linking hypothesis led to different conclusions about the rate of computed implicatures in the experimental task.

### 2.1. Methods

#### 2.1.1. Participants

Two hundred participants were recruited via Amazon Mechanical Turk. They optionally provided demographic information at the end of the study. Participants' mean age was 35. We also asked participants if they had any prior training in logic. 40 participants reported that they did, while 160 had no prior training in logic. All participants' data was included in the final analysis^3^.

#### 2.1.2. Materials and Procedure

**Figure 2:**
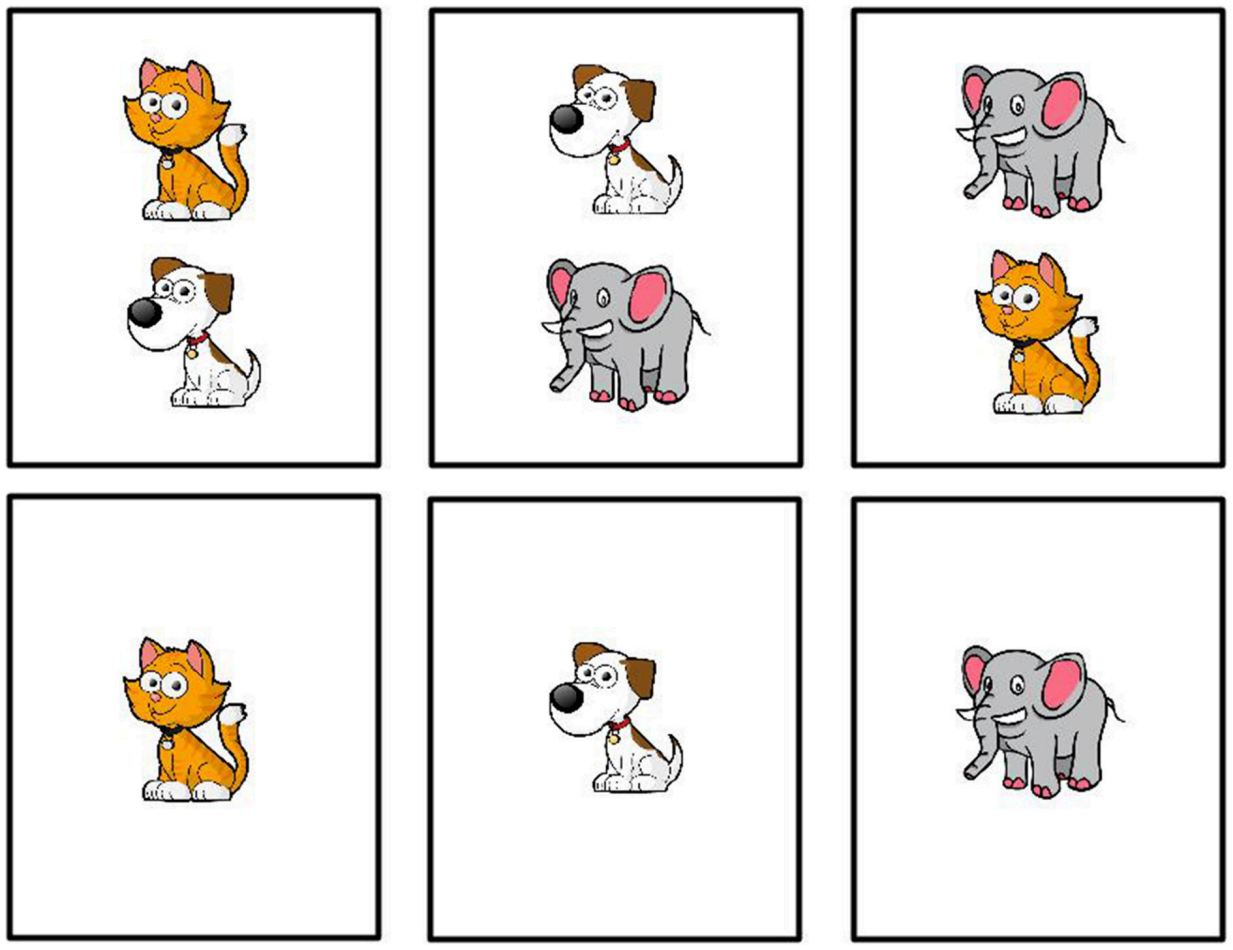
Cards used in the connective guessing game.

The study was administered online through Amazon Mechanical Turk^4^. Participants were first introduced to the set of cards we used in the study (Figure 2). Each card depicted one or two animals, where an animal could be either a cat, a dog, or an elephant. Then participants were introduced to a blindfolded fictional character called Bob. Bob was blindfolded to avoid violations of ignorance expectations associated with the use of disjunction (Chierchia et al., 2001; Sauerland, 2004). Participants were told that Bob would guess the contents of the cards and their task was to indicate whether Bob's guess was wrong or right. On each trial, participants saw a card and a sentence representing Bob's guess. For example, they saw a card with a cat and read the sentence “There is a cat on the card.” They then provided an assessment of Bob's guess. The study ended after 24 trials.

**Figure 3:**
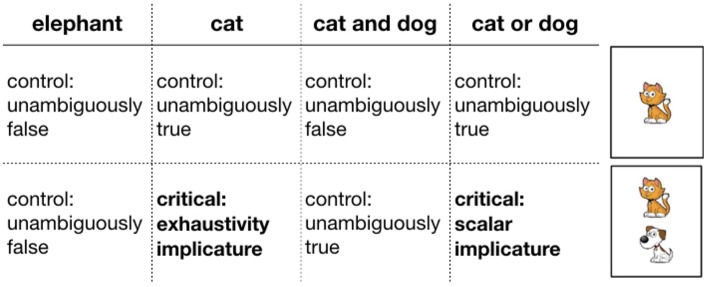
Trial types (critical and control). Headers indicate utterance types. Rows indicate card types. Critical trials are marked in bold.

Two factors were manipulated within participants: card type and guess type. There were two types of cards, cards with only one animal on them and cards with two animals. There were three types of guesses: simple (e.g., *There is a cat*), conjunctive (e.g., *There is a cat and a dog*), and disjunctive (e.g., *There is a cat or a dog*). Crossing card type and guess type yielded trials of varying theoretical interest (see Figure 3): critical underinformative trials that were likely to elicit pragmatic inferences (either scalar or exhaustive) and control trials that were either unambiguously true or false. Each trial type occurred three times with randomly sampled animals and utterances that satisfied the constraint of the trial type. Trial order was randomized.

On critical trials, participants could derive implicatures in two ways. First, on trials on which two animals were present on the card (e.g., cat and dog) but Bob guessed only one of them (e.g., “There is a cat on the card”), the utterance could have a literal interpretation (“There is a cat and possibly another animal on the card”) or an exhaustive interpretation (“There is only a cat on the card”). We refer to these trials as “exhaustive”. Second, on trials on which two animals were on the card (e.g., a cat and a dog) and Bob used a disjunciton (e.g., “There is a cat or a dog on the card”), the utterance could have the literal, inclusive, interpretation, or a pragmatic, exclusive interpretation. We refer to these trials as “scalar.”

In order to assess the effect of the number of response options on implicature rate, we manipulated number of response options in the forced choice task between participants. We refer to the choice conditions as “binary” (options: *wrong, right*), “ternary” (options: *wrong, neither, right*), “quaternary” (options: *wrong, kinda wrong, kinda right, right*), and “quinary” (*wrong, kinda wrong, neither, kinda right, right*). Thus, the endpoint labels always remained the same. If there was an uneven number of response options, the central option was *neither*. Participants were randomly assigned to one of the four task conditions.

### 2.2. Results and Discussion

**Figure 4:**
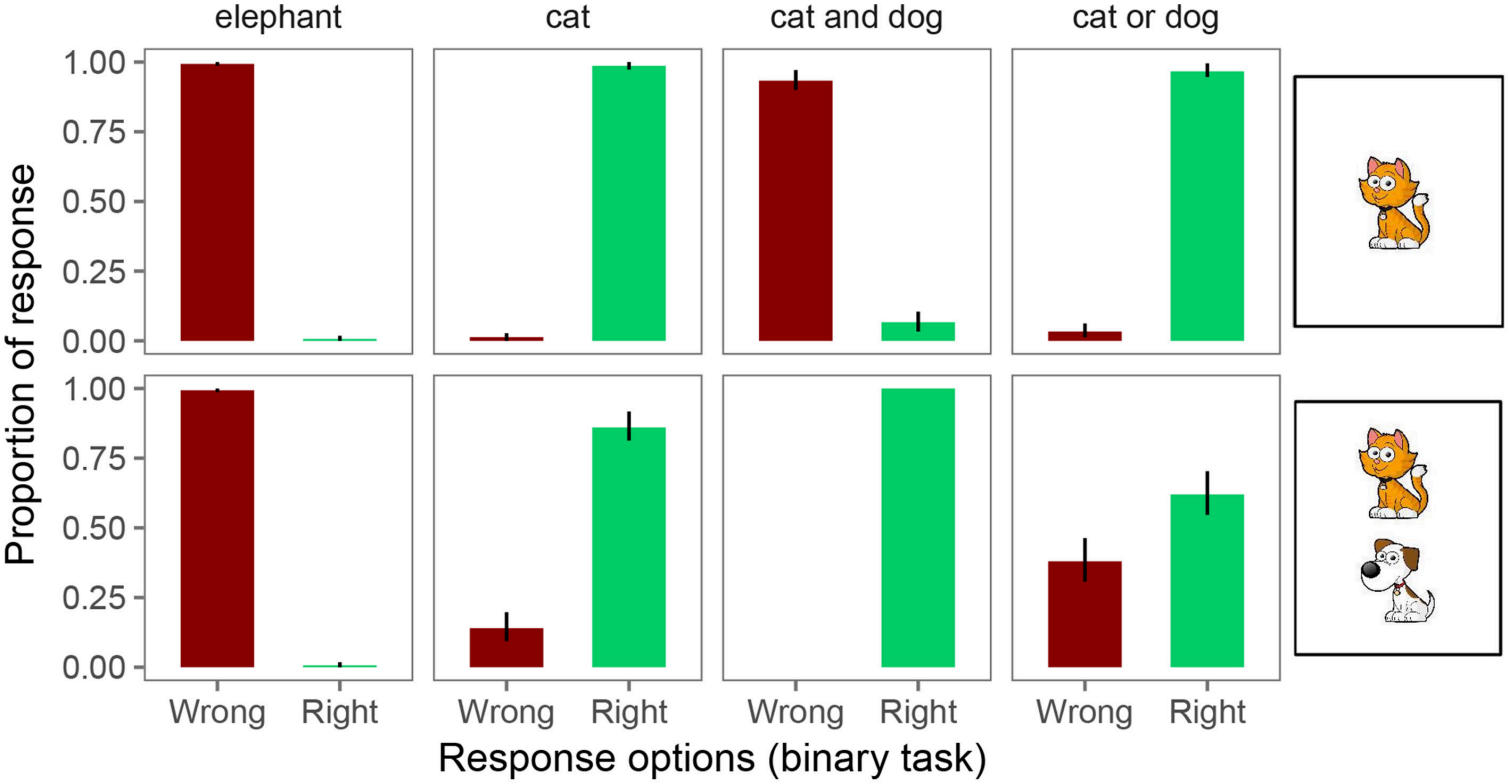
Proportion of responses for the binary forced choice judgments. Error bars indicate 95% binomial confidence intervals (Sison and Glaz, 1995).

**Figure 5:**
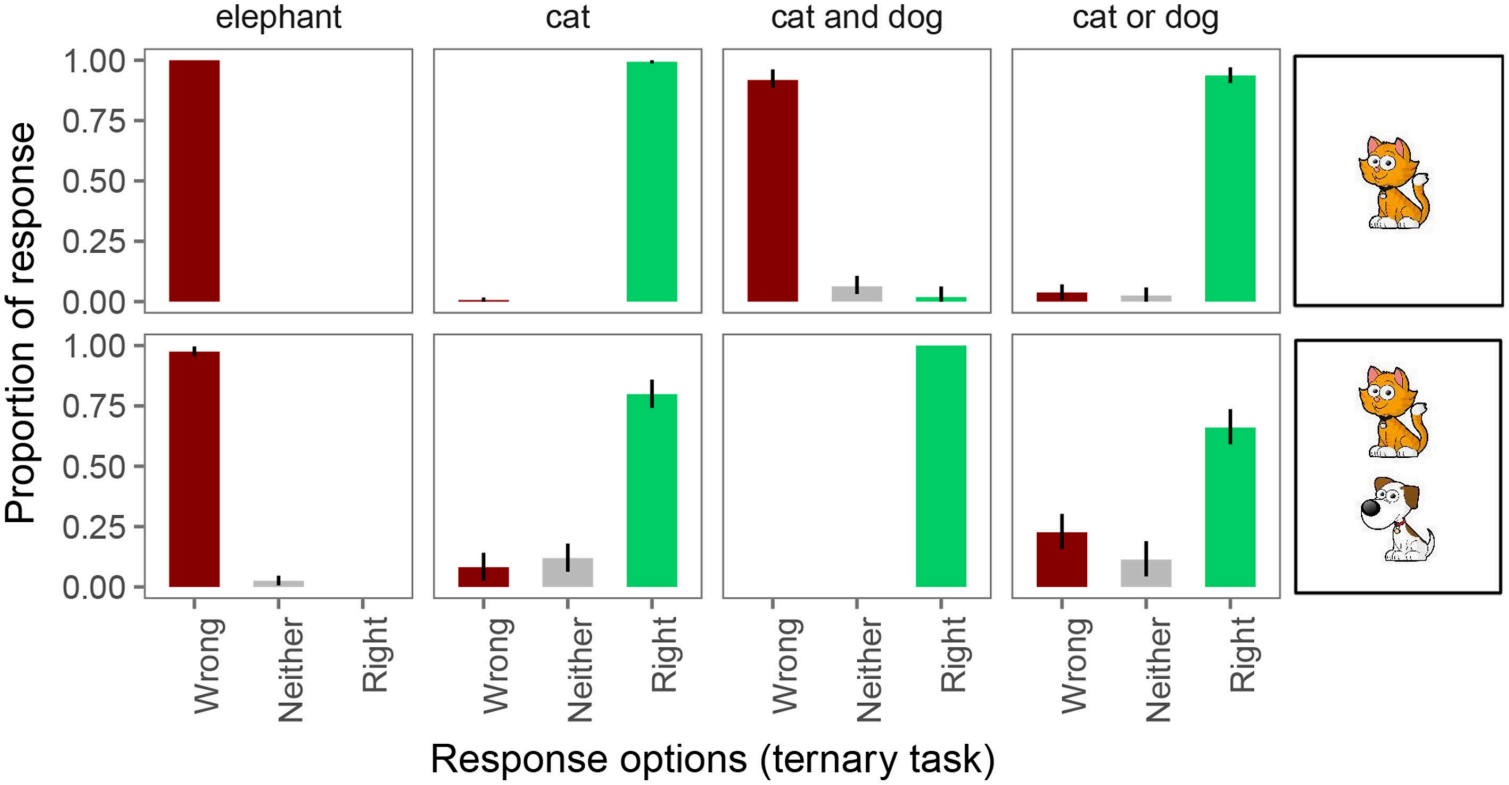
Proportion of responses for the ternary forced choice judgments. Error bars indicate 95% multinomial confidence intervals (Sison and Glaz, 1995).

**Figure 6:**
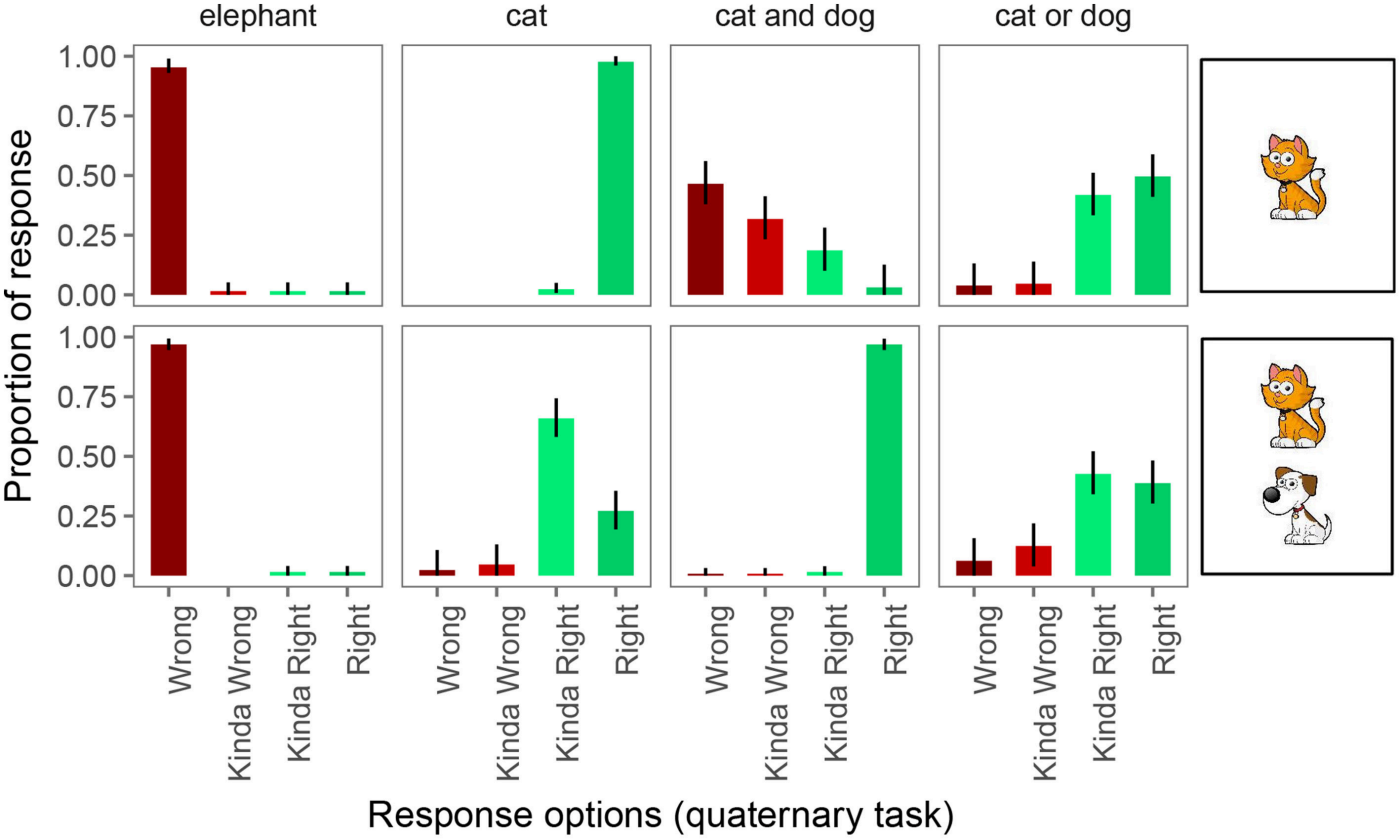
Proportion of responses for the quaternary forced choice judgments. Error bars indicate 95% multinomial confidence intervals (Sison and Glaz, 1995).

**Figure 7:**
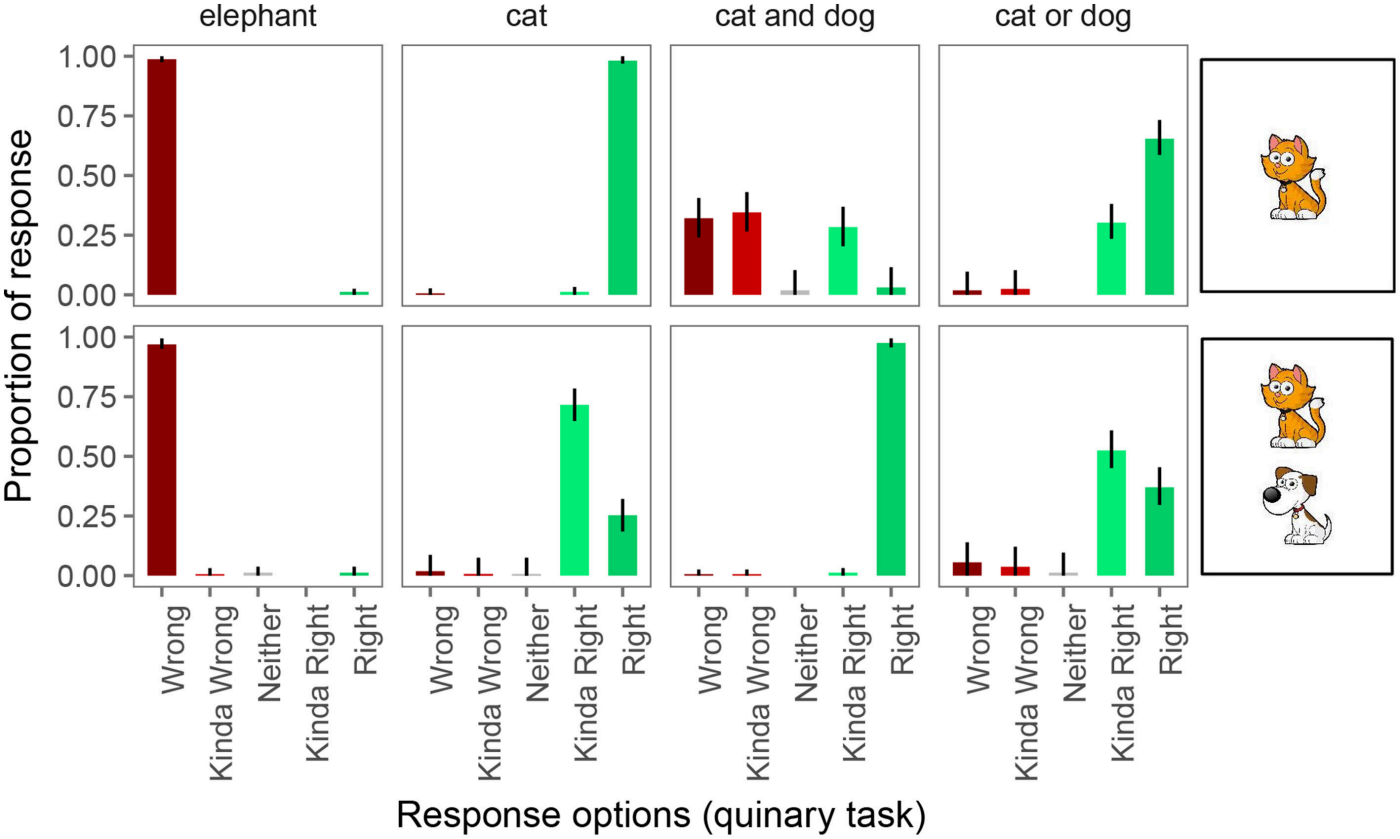
Proportion of responses for the quinary forced choice judgments. Error bars indicate 95% multinomial confidence intervals (Sison and Glaz, 1995).

The collected dataset contains 50 participants in the binary task, 53 in the ternary task, 43 in the quaternary task, and 54 in the quinary task. Figures 4–**7** show the proportions of response choices in each of the 8 trial types on each of the four response tasks, respectively. We report the relevant patterns of results qualitatively before turning to the quantitative analysis of interest.

#### 2.2.1. Qualitative Analysis

In the binary task, participants were at or close to ceiling in responding “right” and “wrong” on unambiguously true and false trials, respectively (see Figure 4). However, on underinformative trials (i.e., a “cat” or “cat or dog” description for a card with both a cat and a dog), we observe pragmatic behavior: on exhaustive trials, participants judged the utterance “wrong” 14% of the time; on scalar trials, participants judged the utterance “wrong” 38% of the time. That is, both under the Weak and Strong link assumptions introduced in the Introduction, inferred implicature rate on exhaustive trials is 14% and on scalar trials 38%.

In the ternary task, participants were also at or close to ceiling in responding “right” and “wrong” on unambiguously true and false trials, respectively (see Figure 5). And again, on underinformative trials (a “cat” and “cat or dog” description for a card with both a cat and a dog), we observed pragmatic behavior: on exhaustive trials, participants considered the guess “wrong” 8% of the time and neither wrong nor right 12% of the time. On scalar trials, participants judged the guess “wrong” 23% of the time and “neither” 11% of the time. This means that the Weak and Strong link lead to different conclusions about implicature rates on the ternary task. Under the Weak link, inferred implicature rate on exhaustive trials is 20%; under the Strong link it is only 8%. Similarly, under the Weak link, inferred implicature rate on scalar trials is 34%; under the Strong link it is only 23%.

In the quaternary task (Figure 6), participants were again at or close to ceiling in responding “right” and “wrong” on 4 of the 6 unambiguously true and false trials. However, with four response options, two of the control conditions appear to be showing signs of pragmatic infelicity: when a conjunction was used and only one of the animals was on the card, participants considered the guess “wrong” most of the time (46%), but they often considered it “kinda wrong” (32%) or even “kinda right” (19%). This suggests that perhaps participants considered the notion of a partially true or correct statement in our experimental setting. Disjunctive descriptions of cards with only one animal, while previously at ceiling for “right” responses, were downgraded to only “kinda right” 26% of the time, presumably because these utterances are also underinformative, though the degree of underinformativeness may be less egregious than on scalar trials.

On underinformative exhaustive trials, we observed pragmatic behavior as before: participants judged the guess “wrong” 2% of the time, “kinda wrong” 5% of the time, and “kinda right” 66% of the time. On scalar trials, participants judged the guess “wrong” 6% of the time, “kinda wrong” 12% of the time, and “kinda right” 43% of the times.

Thus, we are again forced to draw different conclusions about implicature rates depending on whether we assume the Weak link or the Strong link. Under the Weak link, inferred implicature rate on exhaustive trials is 73%; under the Strong link it is only 2%. Similarly, under the Weak link, inferred implicature rate on scalar trials is 61%; under the Strong link it is only 6%.

Finally, Figure 7 shows the proportion of responses in the quinary task. Performance on the 4 pragmatically felicitous control trials was again at floor and ceiling, respectively. The 2 control conditions in which the quaternary task had revealed pragmatic infelicity again displayed that pragmatic infelicity in the quinary task, suggesting that this is a robust type of pragmatic infelicity that, nonetheless, requires fine-grained enough response options to be detected experimentally.

On underinformative exhaustive trials, we observed pragmatic behavior as before: participants judged the guess “wrong” 2% of the time, “kinda wrong” 1% of the time, “neither” 1% of the time, and “kinda right” 72% of the time. On scalar trials, participants judged the guess “wrong” 6% of the time, “kinda wrong” 4% of the time, “neither” 1% of the time, and “kinda right” 52% of the time.

Thus, we would again draw different conclusions about implicature rates depending on whether we assume the Weak link or the Strong link. Under the Weak link, inferred implicature rate on exhaustive trials is 76%; under the Strong link it is only 2%. Similarly, under the Weak link, inferred implicature rate on scalar trials is 63%; under the Strong link it is only 6%.

#### 2.2.2. Quantitative Analysis

**Figure 8:**
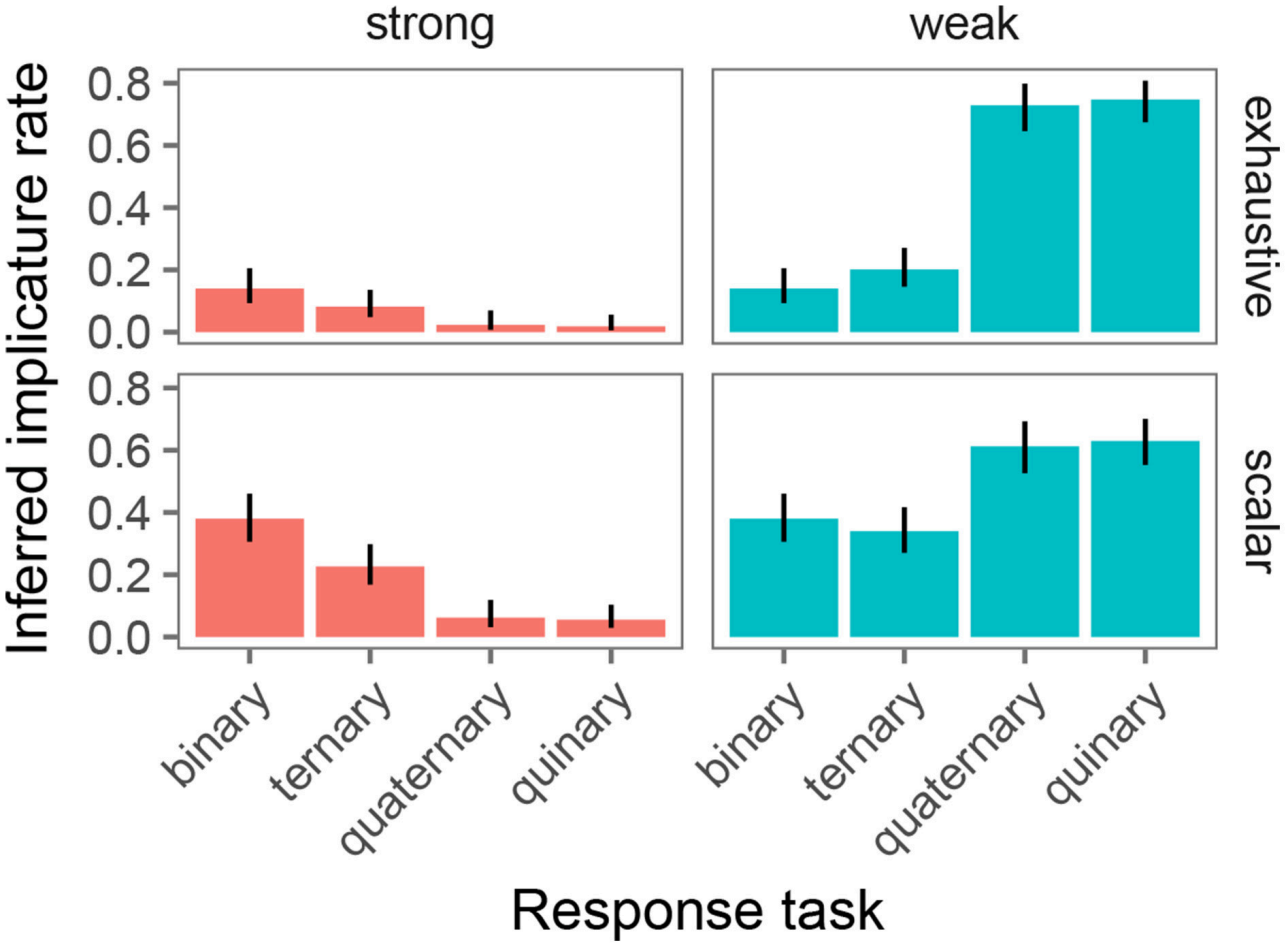
Inferred implicature rates on exhaustive and scalar trials as obtained with the binary, ternary, quaternary, and quinary response task. Columns indicate link from response to implicature rate (strong: proportion of “wrong judgments; weak: proportion of non-right” judgments). Error bars indicate 95% binomial confidence intervals.

Our primary goal in this study was to test whether the estimated implicature rate in the experimental task is affected by the linking hypothesis and the number of response options available to participants. To this end, we only analyzed the critical trials (exhaustive and scalar). In particular, we classified each data point from critical trials as constituting an implicature (1) or not (0) under the Strong and Weak link. Figure 8 shows the resulting implicature rates by condition and link. It is immediately apparent that there is variability in inferred implicature rate. In particular, the Weak link appears to result in greater estimates of implicature rates in tasks with four or five response options, compared to the Strong link. For the binary and ternary task, the assumed link appears to play a much smaller role.

To analyze the effect of link and response options on inferred implicature rate, we used a Bayesian binomial mixed effects model using the R packge “brms” (Bürkner, 2016) with weakly informative priors.^5^ The model predicted the log odds of implicature over no implicature from fixed effects of *response type* (binary, ternary, quaternary, quinary—dummy-coded with binary as reference level), *link* (strong vs. weak—dummy-coded with strong as reference level), and trial type (exhaustive vs. scalar—dummy-coded, with exhaustive as reference level), as well as their two-way and three-way interactions. Following Barr et al. (2013), we included the maximal random effects structure justified by the design: random intercepts for items (cards) and participants, random by-participant slopes for link, trial type, and their interaction, and random by-item slopes for link, trial type, response type, and their interactions. Since the number of response options was a between-participant variable we did not include random slopes of response options for participants. Four chains converged after 2,000 iterations each (warmup = 1,000). Table 1 summarizes the mean parameter estimates and their 95% credible intervals. $\hat{R}=1$ for all estimated parameters. All the analytical decisions described here were pre-registered^6^.

**Table 1:** Model parameter estimates and their credible intervals.

**Predictors**	**Estimate**	**2.5%**	**97.5%**	**Evidence**
Intercept	−8.60	−13.98	−4.53	^*^
Link = Weak	−0.15	−4.86	4.77	
Task = Quaternary	−1.83	−8.08	4.20	
Task = Quinary	−4.05	−10.90	2.38	
Task = Ternary	−1.45	−7.31	4.56	
Implicature = Scalar	6.09	1.00	12.29	^*^
Link = Weak : Task = Quaternary	14.03	7.24	21.88	^*^
Link = Weak : Task = Quinary	17.28	10.64	25.80	^*^
Link = Weak : Task = Ternary	3.81	−1.49	9.22	
Link = Weak : Implicature = Scalar	0.90	−4.01	6.43	
Task = Quaternary : Implicature = Scalar	−5.67	−13.66	1.54	
Task = Quinary : Implicature = Scalar	−2.31	−9.30	4.61	
Task = Ternary : Implicature = Scalar	−1.31	−7.70	4.65	
Link=Weak : Task=Quaternary : Implicature=Scalar	−3.29	−12.07	4.55	
Link=Weak : Task=Quinary : Implicature=Scalar	−7.74	−16.59	−0.16	^*^
Link=Weak : Task=Ternary : Implicature=Scalar	−1.44	−7.00	4.22	

*Rows marked with an asterisk in the evidence column do not contain 0 in the credible interval, thereby providing evidence for an effect*.

The model provided evidence for the following effects: First, there was a main effect of trial type such that scalar trials resulted in greater implicature rates than exhaustive trials (Mean Estimate = 6.09, 95% Credible Interval=[1, 12.29]). Second, there was an interaction between link and number of response options such that the quaternary task (Mean Estimate = 14.03, 95% Credible Interval = [7.24, 21.88]) and the quinary task (Mean Estimate = 17.28, 95% Credible Interval = [10.64, 25.80]) resulted in greater implicature rates with a weak link than with a strong link, but there was no evidence of a link-dependent difference in inferred implicature rate for the binary and ternary task. Finally, there was a three-way interaction between link, trial type, and number of response options, driven by the binary/quinary contrast (Mean Estimate = −7.74, 95% Credible Interval=[−16.59, −0.16]). Simple effects analysis on only the binary and quinary trials, separately for the exhaustive and scalar subset of the data, revealed that the three-way interaction is driven by a different effect of number of response options under the Weak vs. Strong link for the two inference types. Specifically, on exhaustive trials, number of response options (2 vs. 5) only resulted in greater implicature rates under the Weak (β = .2, *p* < 0.0001), but not the Strong link (β = −0.8, *p* < .82). In contrast, on scalar trials, number of response options (2 vs. 5) resulted in greater implicature rates under the Weak (β = 3.6, *p* < 0.005) link, and in lower implicature rates under the Strong link (β = -4.0, *p* < 0.0007).

In sum, both number of response options and link affected the inferred implicature rate, as did the type of inference (exhaustive vs. scalar).

## 3. General Discussion

### 3.1. Summary and Methodological Discussion

In this paper we asked whether linking hypothesis and number of response options available to participants in truth value judgment tasks affects inferred implicature rates. The results presented here suggest they do. A linking assumption that considered the highest point on the scale literal and any lower point pragmatic (Weak link) resulted in higher implicature rates in tasks with 4 or 5 response options compared to the standard two options. A linking hypothesis that considered the lowest point on the scale pragmatic and any higher point literal (Strong link) reported lower implicature rates in tasks with 4 or 5 options compared to the standard two options. The results suggest that the choice of linking hypothesis is a crucial analytical step that can significantly impact the conclusions drawn from truth value judgment tasks. In particular, there is danger for pragmatic ability to be both under- and overestimated.

While the binary truth value judgement task avoids the analytic decision between Strong and Weak linking hypothesis, the results reported here suggest that binary tasks can also underestimate participants' pragmatic competence. In binary tasks, participants are often given the lowest and highest points on a scale (“wrong” vs. “right”) and are asked to report pragmatic infelicities using the lowest point (e.g., “wrong”). The study reported here showed that on trials with true but pragmatically infelicitous descriptions, participants often avoided the lowest point on the scale if they were given more intermediate options. Even though the option “wrong” was available to participants in all tasks, participants in tasks with intermediate options chose it less often. In computing implicature rate, this pattern manifested itself as a decrease in implicature rate under the Strong link when more response options were provided, and an increase in implicature rate under the Weak link when more response options were provided. These observations are in line with Katsos and Bishop (2011)'s argument that pragmatic violations are not as severe as semantic violations and participants do not penalize them as much. Providing participants with only the extreme ends of the scale (e.g., wrong/right, false/true) when pragmatic violations are considered to be of an intermediate nature risks misrepresentation of participants' pragmatic competence. It further suggests that in studies that use binary tasks to investigate response-contingent processing, proportions of “literal” responses may be a composite of both literal and pragmatic underlying interpretations that just happen to get mapped differently onto different response options by participants.

This study did not investigate the effect of response labels on the inferred implicature rate. However, the results provided suggestive evidence that some options better capture participant intuitions of pragmatic infelicities than others. Among the intermediate options, “kinda right” was chosen most often to report pragmatic infelicities. The option “neither” was rarely used in the ternary and quinary tasks (where it was used as a midpoint), suggesting that participants interpreted pragmatic infelicities as different degrees of being “right” and not “neither right nor wrong.” Therefore, options that capture degrees of being “right” like “kinda right” may prove most suitable for capturing infelicity in the long run. We leave this as a methodological issue for future research.

The study had three further design features worth investigating in future work. First, the utterances were ostensibly produced by a blindfolded character. This was an intentional decision to control for violation of ignorance expectations with disjunction. A disjunction such as “A or B” often carries an implication or expectation that the speaker is not certain which alternative actually holds. Future work should investigate how the violation of the ignorance expectation interacts with link and number of response options in inferred implicature rate. Second, in this study we considered exhaustive and scalar implicatures with *or*. If the observed effects of link and number of response options hold in general, they should be observable using other scales, e.g., on implicatures with *some*. Finally, our experiment was designed as a guessing game and the exact goal or task-relevant Question Under Discussion of the game was left implicit. Given the past literature on QUD effects on scalar implicature, we expect that different goals—e.g., to help the character win more points vs. to help the character be more accurate—would affect how strict or lenient participants are with their judgments and ultimately affect implicature rate in the task (Zondervan, 2010; Degen and Goodman, 2014). Future work should systematically vary the goal of the game and explore its effects on the inferred implicature rate. But crucially, it's unlikely that the observed effects of number of response options and linking hypothesis on inferred implicature rate are dependent on any of the discussed design choices.

### 3.2. Revisiting the Linking Hypothesis

On the traditional view of the link between implicature and behavior in sentence verification tasks, scalar implicature is conceptualized as a binary, categorical affair – that is, an implicature is either “calculated” or it isn't, and the behavioral reflexes of this categorical interpretation process should be straightforwardly observed in experimental paradigms. This assumption raises concerns for analyzing variation in behavior on a truth value judgment task; for example, why did the majority of respondents in the binary condition of our experiment answer “right” to an utterance of the underinformative “There is a cat or dog” when the card had both a cat and a dog on it? And why did a sizeable minority nonetheless choose “wrong” in this same condition?

To explain these data on the traditional view, we are forced to say that a) not all participants calculated the implicature; or that b) some participants who calculated the implicature did not choose the anticipated (i.e., “wrong”) response due to some other cognitive process which overrode the “correct” implicature behavior; or some mixture of (a) and (b). We might similarly posit that one or both of these factors underlie the variation in the ternary, quaternary, and quinary conditions. However, without an understanding of how to quantitatively specify the link between implicature calculation and its behavioral expression, the best we can hope for on this approach is an analysis which predicts general qualitative patterns in the data (e.g., a prediction of relatively more “right” responses than “wrong” responses in a given trial of our binary truth value judgment task, or a prediction of a rise in the rate of “right”/“wrong” responses between two experimental conditions, given some contextual manipulation). However, we should stress that to the best of our knowledge, even a qualitative analysis of this kind of variation in behavior on sentence verification tasks – much less the effect of the number of response choices on that behavior – is largely underdeveloped in the scalar implicature literature.

We contrast the above view of implicature and its behavioral reflexes with an alternative linking hypothesis. Recent developments in the field of probabilistic pragmatics have demonstrated that pragmatic production and comprehension can be captured within the Rational Speech Act (RSA) framework (Frank and Goodman, 2012; Degen et al., 2013, 2015; Goodman and Stuhlmüller, 2013; Kao et al., 2014; Qing and Franke, 2015; Bergen et al., 2016; Franke and Jäger, 2016; Goodman and Frank, 2016). Much in the spirit of Gricean approaches to pragmatic competence, the RSA framework takes as its point of departure the idea that individuals are rational, goal-oriented communicative agents, who in turn assume that their interlocutors similarly behave according to general principles of cooperativity in communication. Just as in more traditional Gricean pragmatics, pragmatic inference and pragmatically-cooperative language production in the RSA framework are, at their core, the product of counterfactual reasoning about alternative utterances that one might produce (but does not, in the interest of cooperativity). However, the RSA framework explicitly and quantitatively models cooperative interlocutors as agents whose language production and comprehension is a function of Bayesian probabilistic inference regarding other interlocutors' expected behavior in a discourse context.

Specifically, in the RSA framework we model pragmatically competent listeners as continuous probabilistic distributions over possible meanings (states of the world) given an utterance which that listener observes. The probability with which this listener *L*_1_ ascribes a meaning *s* to an utterance *u* depends upon a prior probability distribution of potential states of the world *P*_*w*_, and upon reasoning about the communicative behavior of a speaker *S*_1_. *S*_1_ in turn is modeled as a continuous probabilistic distribution over possible utterances given an intended state of the world the speaker intends to communicate. This distribution is sensitive to a rationality parameter α, the production cost *C* of potential utterances, and the informativeness of the utterance, quantified via a representation of a literal listener *L*_0_ whose interpretation of an utterance is in turn a function of that utterance's truth conditional content [[*u*]](*s*) and her prior beliefs about the state of the world *P*_*w*_(*s*).

$$\begin{array}{l} P_{L_{1}}\left(s|u\right)\propto P_{S_{1}}\left(u|s\right)*P_{w}\left(s\right)\\ P_{S_{1}}\left(u|s\right)\propto exp\left(\alpha \left(log\left(P_{L_{0}}\left(s|u\right)\right)-C\left(u\right)\right)\right)\\ P_{L_{0}}\left(s|u\right)\propto \left[\left[u\right]\right]\left(s\right)*P_{w}\left(s\right) \end{array}$$

This view contrasts with the traditional view in that it is rooted in a quantitative formalization of pragmatic competence which provides us a continuous measure of pragmatic reasoning. In the RSA framework, individuals never categorically draw (or fail to draw) pragmatic inferences about the utterances they hear. For example, exclusivity readings of disjunction are represented in RSA as relatively lower posterior conditional probability of a conjunctive meaning on the *P*_*L*_ distribution given an utterance of “or”, compared to the prior probability of that meaning. Thus, absent auxiliary assumptions about what exactly would constitute “implicature,” it is not even possible to talk about rate of implicature calculation in the RSA framework. The upshot, as we show below, is that this view of pragmatic competence does allow us to talk explicitly and quantitatively about rates of observed behavior in sentence verification tasks.

We take inspiration from the RSA approach and treat participants' behavior in our experimental tasks as the result of a soft-optimal pragmatic speaker in the RSA framework. That is, following Degen and Goodman (2014), we proceed on the assumption that behavior on sentence verification tasks such as truth value judgment tasks, is best modeled as a function of an individual's mental representation of a cooperative speaker (*S*_1_ in the language of RSA) rather than of a pragmatic listener who interprets utterances (*P*_*L*_1__)^7^. In their paper, Degen and Goodman show that sentence verification tasks are relatively more sensitive to contextual features like the Question Under Discussion than are sentence interpretation tasks, and that this follows if sentence interpretation tasks—but not sentence verification tasks—require an additional layer of counterfactual reasoning about the intentions of a cooperative speaker.

A main desideratum of a behavioral linking hypothesis given the RSA view of pragmatic competence is to transform continuous probability distributions into categorical outputs (e.g., responses of “right”/“wrong” in the case of the binary condition of our experiment). For a given utterance *u* and an intended communicated meaning *s*, *S*_1_(u | s) outputs a conditional probability of *u* given *s*. For example, in the binary condition of our experiment where a participant evaluated “There is a cat or a dog” when there were both animals on the card, the participant has access to the mental representation of *S*_1_ and hence to the *S*_1_ conditional probability of producing the utterance “cat or dog” given a cat and dog card: *S*_1_(“cat or dog” | cat and dog). According to the linking hypothesis advanced here, the participant provides a particular response to *u* if the RSA speaker probability of *u* lies within a particular probability interval. We model a responder, *R*, who in the binary condition responds “right” to an utterance *u* in world *s* just in case *S*_1_(*u*|*s*) meets or exceeds some probability threshold θ:

$$\begin{array}{l} \text{ R}\left(\text{u},\text{w},\theta \right)\\ =\text{“right” iff }S_{1}\left(\text{u}|s\right)\geq \theta \\ =\text{“wrong” otherwise} \end{array}$$

The model of a responder in the binary condition is extended intuitively to the condition where participants had three response options. In this case, we allow for two probability thresholds: θ_1_, the minimum standard for an utterance in a given world state to count as “right”, and θ_2_, the minimum standard for “neither”. Thus, in the ternary condition, R(u, s, θ_1_, θ_2_) is “right” iff *S*_1_(u | s) ≥ θ_1_ and “neither” iff θ_1_ > *S*_1_(u | s) ≥ θ_2_. To fully generalize the model to our five experimental conditions, we say that *R* takes as its input an utterance *u*, a world state *s*, and a number of threshold variables dependent on a variable *c*, corresponding to the experimental condition in which the participant finds themself (e.g., the range of possible responses available to *R*).

Given c = “ternary”
R(u, w, θ_1_, θ_2_)
= “right” iff *S*_1_(u | s) ≥ θ_1_
= “neither” iff θ_1_ > *S*_1_(u | s) ≥ θ_2_
= “wrong” otherwise
Given c = “quaternary”
R(u, w, θ_1_, θ_2_, θ_3_)
= “right” iff *S*_1_(u | s) ≥ θ_1_
= “kinda right” iff θ_1_ > *S*_1_(u | s) ≥ θ_2_
= “kinda wrong” iff θ_2_ > *S*_1_(u | s) ≥ θ_3_
= “wrong” otherwise
Given c = “quinary”
R(u, w, θ_1_, θ_2_, θ_3_. θ_4_)
= “right” iff *S*_1_(u | s) ≥ θ_1_
=“kinda right” iff θ_1_ > *S*_1_(u | s) ≥ θ_2_
= “neither” iff θ_2_ > *S*_1_(u | s) ≥ θ_3_
= “kinda wrong” iff θ_3_ > *S*_1_(u | s) ≥ θ_4_
= “wrong” otherwise

In an RSA model, *S*_1_(u | s) will be defined for any possible combination of possible utterance and possible world state. One consequence of this is that for the purposes of our linking hypothesis, participants are modeled as employing the same decision criterion – does *S*_1_(u | s) exceed the threshold? – in both “implicature” and “non-implicature” conditions of a truth value judgment task experiment. That is, participants never evaluate utterances directly on the basis of logical truth or falsity: for example, our blindfolded character Bob's guess of “cat and dog” on a cat and dog card trial is “right” to the vast majority of participants not because the guess is logically true but because *S*_1_(“cat and dog” | cat and dog) is exceedingly high.

**Figure 9:**
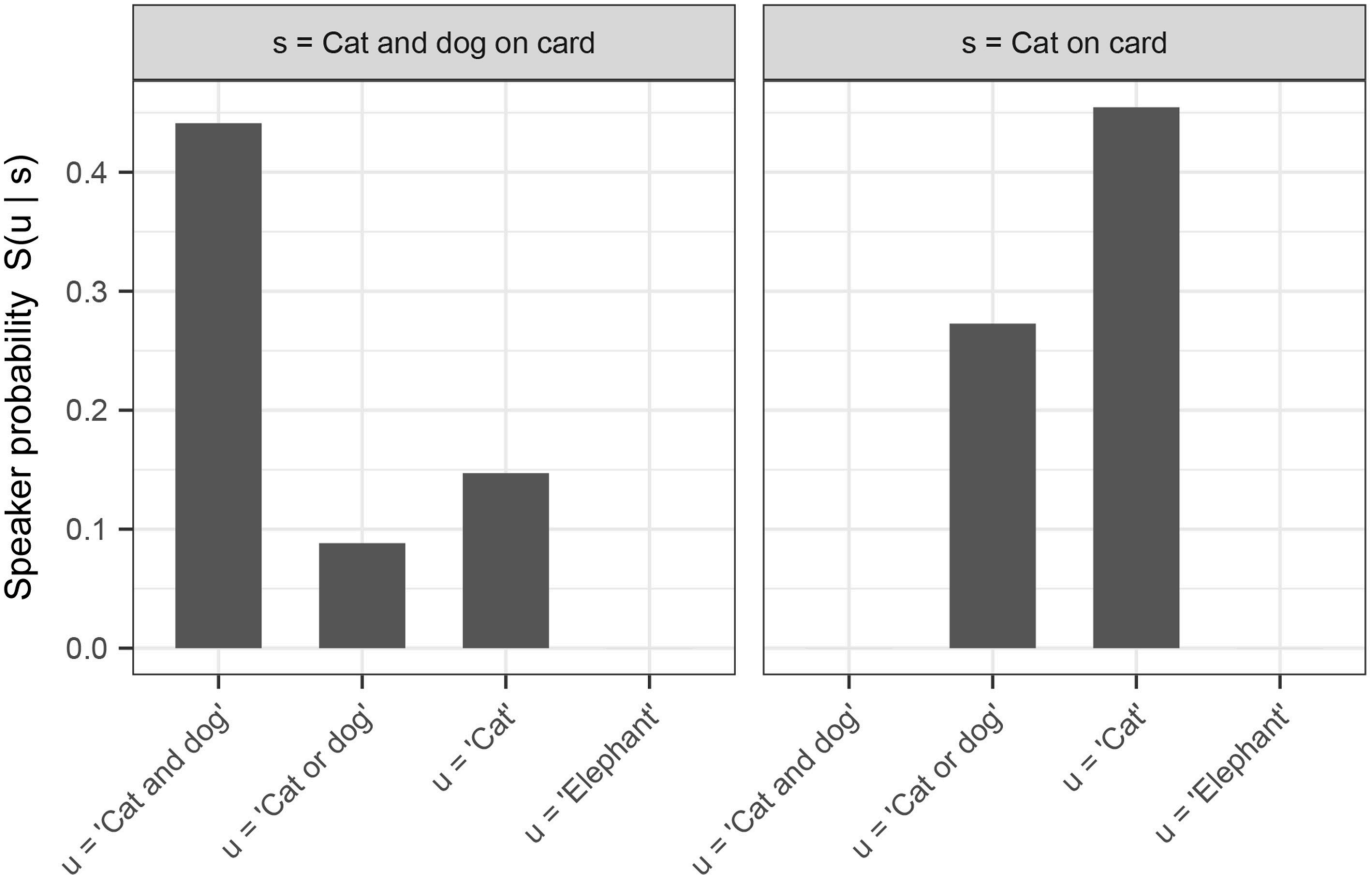
Speaker probabilities of utterances on the exhaustive and scalar trials, as obtained using the model described in this section.

For further illustration, we use our definition of a pragmatically-competent speaker *S*_1_ (as defined above) to calculate the speaker probabilities of utterances in states of the world corresponding to our experimental conditions (i.e., for “cat,” “dog,” “cat and dog,” and “elephant,” given either a cat on the card, or both a cat and a dog on the card). In calculating these probabilities, we assume that the space of possible utterances is the set of utterances made by Bob in our experiment (i.e., any possible single, disjunctive, or conjunctive guess involving “cat,” “dog,” or “elephant”). For the purposes of our model, we assume a uniform cost term on all utterances. We furthermore assume that the space of possible meanings corresponds to the set of possible card configurations that a participant may have seen in our experiment, and that the prior probability distribution over these world states is uniform. Lastly, we set α—the speaker rationality parameter—to 1. The resulting speaker probabilities are shown in Figure 9.^8^

The linking hypothesis under discussion assumes that speaker probabilities of utterance given meaning are invariant across a) our four different experimental conditions, b) across participants, and c) within participants (that is, participants do not update their *S*_1_ distribution in a local discourse context). We note that the assumption (b) may conceivably be relaxed by allowing one or more of the parameters in the model – including the prior probability over world states *P*_*w*_, the cost function on utterances *C*, or the rationality parameter α—to vary across participants. We also note that assumption (c) in particular is in tension with a growing body of empirical evidence that semantic and pragmatic interpretation is modulated by rapid adaptation to the linguistic and social features of one's interlocutors (Fine et al., 2013; Kleinschmidt and Jaeger, 2015; Yildirim et al., 2016).

However, if we should like to keep the above simplifying assumptions in place, then this linking hypothesis commits us to explaining variation in the data in terms of the threshold parameters of our responder model *R*. Consider first the variation in response across different experimental conditions on a given trial, e.g., evaluation of a guess of “cat and dog” when the card contains both a cat and a dog. The variation in the proportion of responses of “right” on this trial between the binary, ternary, quaternary, and quinary conditions indicates that the threshold value for “right” responses must vary across conditions; that is, we predict that the θ of the binary condition will differ from, e.g., the θ_1_ of the ternary condition as well as the θ_1_ of the quaternary condition. We also observed variation in response on this trial within a single condition (for example, a sizeable minority of participants responded “wrong” to this trial in the binary condition). Thus, this linking hypothesis is committed to the notion that threshold values may vary across participants, such that a speaker probability of utterance *S*_1_(u | s) can fall below θ for some subset of participants while *S*_1_(u | s) itself remains constant across participants.

Lastly, for two utterances of the same conditional probability and in the same experimental condition, participants in our experiment sometimes provided a judgment of “right” to one utterance but “wrong” to the other. That is, there was within-subject variation in this experiment. One way to represent such variation would be to posit that the parameterization of threshold values proceeds stochastically and that threshold values are recalibrated for every individual sentence verification task. Rather than representing a threshold as a discrete value N between 0 and 1, we can represent that threshold as a distribution over possible threshold values – with mass centered around N. Whenever an individual encounters a single trial of our truth value judgment task experiment, a threshold value is sampled from this distribution. By allowing values of θ to vary stochastically in this way, we can capture that *S*_1_(u | s) can fall both above and below θ for a given participant.

The model in its present form already captures an interesting asymmetry in inferred implicature rates between exhaustive and scalar trials of the experiment: note specifically (c.f. Figure 8) that inferred implicature rates are greater in the binary and ternary conditions for scalar trials over exhaustive trials. This is expected given the model's inferred speaker probabilities: the speaker probability of producing “There is a cat on the card” in the context of there being a cat and dog on the card (an exhaustive implicature-inducing trial) is greater than the speaker probability of producing “There is a cat or a dog on the card” in that same context (a scalar implicature-inducing trial). Assuming noisy θ values centered around N, participants are expected to respond “Right” more frequently on exhaustive than on scalar trials, which is precisely what is observed. Recall that these probabilities were derived via the simplifying assumption of uniform cost on utterances; in fact, adding cost to relatively complex disjunctive sentences over simple declarative sentences only predicts a more pronounced asymmetry in the experimentally-observed direction.

As suggested above, the quantitative predictions of our model will depend crucially on the values assigned to its free parameters - including (but not limited to) the probability thresholds and speaker costs of possible utterances. However, the values of these parameters can be estimated in a principled and informed manner through Bayesian statistical analysis of our experimental data. Samples from prior distributions over possible parameter values yield predicted patterns of response, which are then compared against empirically-observed response patterns in order to determine the a posteriori probability that these values are in fact the “real” latent parameter values. The resulting posterior distributions are sampled from in turn, in order to parameterize the model and assess overall quantitative fit given the data. Though we leave a quantitative assessment of our model to future work, we sketch the general procedure here to emphasize that the model is amenable to rigorous and data-driven evaluation.

One empirical problem is the pattern of responses we observed for “cat and dog” on trials where there was only a cat on the card. Because this utterance is strictly false in this world state, it is surprising—on both the traditional view as well as on the account developed here—that participants assigned this utterance ratings above “wrong” with any systematicity. However, this is what we observed, particulary in the quaternary and quinary conditions of the experiment, where a sizeable minority of participants considered this utterance “kinda right”. As Figure 9 demonstrates, the conditional speaker probability of this utterance in this world state is 0; thus, there is no conceivable threshold value that would allow this utterance to ever be rated above “wrong” (on the reasonable assumption that the thresholds in our responder model *R* should be non-zero). Any linking hypothesis will have to engage with this data point, and we leave to future work an analysis which captures participants' behavior in this condition.

For the time being, however, we present the above analysis as a proof of concept for the following idea: by relaxing the assumptions of the traditional view of scalar implicature—namely, that scalar implicatures either are or are not calculated, and that behavior on sentence verification tasks directly reflects this binary interpretation process—we can propose quantitative models of the variation in behavior that is observed in experimental settings. We note that the linking hypothesis proposed here is just one in the space of possible hypotheses. For example, one might reject this threshold-based analysis in favor of one whereby responses are the outcomes of sampling on the (pragmatic speaker or pragmatic listener) probability distributions provided by an RSA model. We leave this systematic, quantitative investigation to future work. For now we emphasize that explicit computational modeling of behavioral responses is a tool that is available to researchers in experimental pragmatics. While using the RSA framework as the modeling tool requires revising traditional assumptions about the nature of scalar implicature by relaxing the crisp notion of scalar implicature as something that is or is not “calculated” in interpretation, it provides new flexibility to explicitly discuss behavior in experimental settings. One need not adopt the RSA framework as the tool for hypothesizing and testing the link between theoretical constructs and behavior in pragmatic experiments. However, the empirical findings we have reported here—that the inferences researchers draw about “implicature rate” are volatile and depend on various features of the paradigm and the linking hypothesis employed— strongly suggest that experimental pragmatics as a field must engage more seriously with the foundational questions of what we are measuring in the experiments we run.

Concluding, we have shown in this paper that inferred “implicature rate”—a ubiquitous notion in theoretical and experimental pragmatics—as estimated in truth value judgment tasks, depends on both the number of responses participants are provided with as well as on the linking hypothesis from proportion of behavioral responses to “implicature rate”. We further sketched an alternate linking hypothesis that treats behavioral responses as the result of probabilistic reasoning about speakers' likely productions. While a thorough model comparison is still outstanding, this kind of linking hypothesis opens a door toward more systematic and rigorous formulation and testing of linking hypotheses between theoretical notions of interest in pragmatics and behavioral responses in experimental paradigms.

## Author Contributions

All authors contributed to the conception and design of the study. MJ conducted the online survey studies; reported the results and performed the statistical analysis. BW conducted the modeling and wrote the discussion section. JD wrote the theoretical introduction, and contributed to the experimental section and the discussion section and the modeling sections. All authors contributed to manuscript revision, read, and approved the submitted version.

### Conflict of Interest Statement

The authors declare that the research was conducted in the absence of any commercial or financial relationships that could be construed as a potential conflict of interest.

## Footnotes

^1^An open question concerns the extent to which the labeling of points on the scale affects judgments (e.g., “wrong”–“right” vs. “false”–“true” vs. “disagree”–“agree”). Studies vary in the labeling of scale points.

^2^Researchers may vary with respect to which linking hypothesis (Weak vs. Strong, or others) they consider most plausible. Supporters of the Weak link may argue that there are three theoretically motivated categories of judgments: false, true but infelicitous, true and felicitous. Under such an account, it is plausible that false and true+felicitous responses occupy the ends of the false/true scale while true but infelicitous responses occupy the mid portion. On critical trials, participants judge underinformative statements that are true but infelicitous and therefore the mid portion of the scale can provide evidence for implicature computation. However, supporters of the Strong link may argue that if a participant computes an implicature, their response in the task should show the commitment to that interpretation by judging the underinformative utterance as false. Any other response shows that they have not truly computed an implicature. So far, these discussions have remained largely informal. In this paper we stay neutral with respect to the plausibility of each link and only aim to demonstrate the consequences of assuming them.

^3^This study was carried out in accordance with the recommendations of the Common Rule, Federal Office for Human Research Protections. The protocol was approved by the Stanford University IRB 2 (non-medical research). All subjects gave Informed consent, documentation was waived by the IRB.

^4^The experiment can be viewed here https://cdn.rawgit.com/thegricean/si-paradigms/94a590f0/experiments/main/1_methods/online_experiment/connective_game.html

^5^For more information about the default priors of the “brms” package, see the brms package manual.

^6^Our preregistration can be accessed at https://aspredicted.org/tq3sz.pdf

^7^Degen and Goodman (2014) argue that sentence verification is more plausibly construed as a production task rather than as an interpretation task because participants, unlike in natural language comprehension, are provided with the ground truth about the state of the world that a speaker is describing. Thus, participants are in essence being asked to assess the quality of a speaker's utterance. In contrast, Degen and Goodman argue, true interpretation tasks are characterized by the listener inferring what the state of the world is that the speaker is describing, for instance by selecting from one of multiple interpretation options.

^8^Note that the probabilities in each facet don't sum to 1 because the model considers all possible disjunctive, conjunctive, and simple utterances, while we are only visualizing the ones corresponding to the experimental conditions.
